# Young people's overestimation of peer substance use: an exaggerated phenomenon?

**DOI:** 10.1111/j.1360-0443.2011.03680.x

**Published:** 2012-05

**Authors:** Hilde Pape

**Affiliations:** Norwegian Institute for Alcohol and Drug ResearchOslo, Norway

**Keywords:** Alcohol, drugs, methodological problems, norms misperception, validity issues, young people

## Abstract

**Aims:**

This paper queries the notion that young people overestimate peer substance use, asking whether there is robust evidence that such misperceptions are widespread and whether the phenomenon may have been exaggerated in the research literature.

**Method:**

An examination of the research literature was conducted, focusing mainly on studies published since 2000. Some analyses of relevant data on cannabis use from a Norwegian youth survey were also undertaken.

**Results:**

The research in question is characterized by many weaknesses, including low response rates and widespread use of convenience samples, as well as the presence of contextual factors and the use of assessment tools that may have created a bias in favour of ‘demonstrating’ that youth overestimate peer drinking or drug use. Moreover, in some cases, the apparent tendency to hold such misbeliefs may reflect the reality. Further, although most studies conclude that the modal tendency is to overestimate, high levels of underestimation of peer substance use have been reported. There is also suggestive evidence that many youth may have no pre-existing beliefs when responding to items on the issue. Results from the Norwegian youth survey added to this picture.

**Conclusion:**

Young people's tendency to overestimate peer drinking and drug use has been exaggerated, while the uncertainty surrounding the evidence in question has been understated.

## INTRODUCTION

In 1963, Bruun & Hauge [1] reported that young men in the Nordic countries tended to believe that their age-mates consumed more alcohol than themselves. The phenomenon was rediscovered years later in studies of American college students [2], and there is now an extensive research literature supporting the notion that young people hold inflated beliefs about their peers' alcohol consumption [3–6]. They also appear to overestimate the extent to which peers approve of drinking and drunkenness, implying that both injunctive (attitudinal) and descriptive (behavioural) norms are likely to be misperceived. While studies from the United States predominate in the field, research conducted elsewhere indicates that such misbeliefs are widespread across nations and drinking cultures [7–13].

The discrepancy between the actual and the perceived level of alcohol use may apparently be quite substantial. For instance, one recent study on college drinking concluded that ‘as in previous research […], participants estimated that their peers drink about double the amount reported by the participants’ ([14], p. 525). Correspondingly, Perkins *et al*. [15] reported, in a nation-wide US study of college and university students, that 71% overestimated their peers' alcohol consumption. Such misperceived descriptive norms have also been observed in studies of other normatively regulated behaviours, including smoking and illegal drug use [5,6].

The social norms theory was developed in this context [5,16,17]. Applied to alcohol use, it posits that when individuals overestimate peer drinking, they are likely to adjust their own consumption to catch up with the misperceived norm. Alternatively, an extensive drinking pattern may be justified and maintained because individuals believe that ‘everybody’ drinks a lot and approves of heavy drinking. Correcting such misperceptions is assumed to produce behavioural changes and preventive measures based on this assumption has gained substantial popularity [18,19].

The social norms theory and approach prevention have been questioned for various reasons [19–21]. However, one basic issue that has been barely discussed is whether there is, in fact, solid evidence that young people tend to overestimate peer drinking and drug use. A related question is whether the magnitude of such overestimation may have been exaggerated. These are issues pursued by examining the research literature more closely, focusing specifically on assessment strategies and validity issues. Some analyses of data from a large school-based survey were also conducted. The purpose was to add to the literature by exploring the prevalence of ‘I don't know’-answers to a question on cannabis use among age-mates, and to inspect the prevalence of both over- and underestimation of such drug use.

## METHOD

This paper is based mainly on an examination of the research literature. Electronic databases were searched using key words such as ‘norms’, ‘perception’, ‘alcohol’ and ‘drug’ and additional publications were found in the reference lists of identified papers. Priority was given to studies published in peer-reviewed journals since 2000. Studies focusing solely on perceived injunctive norms were excluded.

The empirical analyses were based on data from a survey that was carried out in 2005. The target sample comprised all junior and senior high school students in 16 Norwegian municipalities. There were 89 such schools in the municipalities, of which 85 took part in the survey (response rate: 87%). The analyses were confined to the 15–16-year-olds (*n* = 2830) and to the senior high school students [mean age: 16.9 years, standard deviation (SD) = 1.1] (*n* = 11 158) in the sample. Details about the survey are reported elsewhere [22].

## RESULTS AND DISCUSSION

### Assessing the ‘actual’ level of substance use

Most studies in the field have used data on the respondents' personal drinking or drug-taking to assess the actual prevalence, against which their perception of peer substance use has been compared. However, it goes without saying that estimates of ‘actual’ prevalence rates are always surrounded by uncertainty, among other things because self-reports may be unreliable. For neutral issues the response errors may be more or less random, but an extensive literature review concluded recently that ‘respondents are likely to over-report socially desirable behaviours and to under-report socially undesirable ones’ ([23], p. 878). Hence, one may assume that survey data on underage drinking (which is illegal in the United States), excessive drinking and illicit drug use all tend to be biased downwards.

Indeed, the notion that respondents are inclined to understate their alcohol consumption and to give false negative responses to questions on drug use has generally been supported [24–26]. Such misreporting may include elements of self-deception, and thus be motivated more or less unconsciously. To some extent, observed self–other discrepancies with respect to substance use may therefore reflect the respondents' misperception of their *own* drinking or drug use. Under-reporting may also occur because the respondents conceal the truth intentionally, for instance, because they are suspicious about the confidentiality of their responses [27]. However, it is highly unlikely that their estimates of peers' substance use may be affected by such concerns.

Most studies on misperceived drinking norms are based on samples of students in their late teens or early 20s, but whether the tendency to under-report varies with age is unknown. However, according to Borsari & Muellerleile [28], the phenomenon may not apply to college students because they are unlikely to perceive heavy drinking as socially undesirable. Moreover, their meta-analysis showed that college students' self-reported alcohol consumption corresponded quite well with that reported by collateral informants. However, another recent study clearly indicated there *is* a social desirability bias in survey data on college drinking, implying that the observed consumption level is likely to be deflated [29].

Sample representativeness is another basic issue. Ideally, studies aimed at measuring actual prevalence rates should be based on representative samples with response rates close to maximum. However, as reported in Borsari & Carey's [3] review of the social norms literature, convenience samples are used widely while the response rates for studies based on other samples have generally been modest. In fact, participation rates well below 50% are now fairly common (e.g. [8,12,30–33]). It is true that this may not necessarily pose serious problems, but some studies indicate that there is a positive correlation between non-participation and alcohol use [34–36]. If so, survey participants may be quite right when reporting that the average co-student drinks more than themselves. However, the research on non-response bias in relation to drinking is meagre and the findings have been mixed [37]. One may still assume that the lower the response rate, the greater the risk that the sample differs from the population with respect to drinking.

### The dubious use of ‘friends’ as reference group

Borsary & Carey's [4] meta-analysis indicated that the degree to which youth overestimate others' drinking depends on their proximity to the reference group. While the observed self–other discrepancy was substantial when ‘the others’ referred to a distal group (e.g. ‘other students’), it was generally quite modest with respect to the respondents' friends. Numerous subsequent studies have revealed similar findings (e.g. [12,33,38,39]). However, for various reasons, using ‘friends’ as a target is troublesome.

To ensure comparability between data on ‘actual’ and perceived norms, it is evidently essential that the respondents are representative for the reference group in question. This may not be the case when the comparison target is ‘your friends’. It is possible that a population of students and the corresponding population of ‘students who are somebody's friends’ overlap substantially, but friendship ties are not necessarily symmetrical. In one recent study, more than 20% of those who had been nominated as ‘my best friend’ by a schoolmate did not reciprocate the relationship [40]. Moreover, while some individuals are seen as a friend by numerous people, others are hardly perceived as such by anyone.

If young people who have many friendship ties also tend to drink a great deal, observed self–friend discrepancies with respect to drinking may reflect an actual state of affairs. Several studies indicate that this is, in fact, the case [41–44]. For instance, one of them concluded that ‘socializing with others, having friends and a regular relationship associated independently with drunkenness-related alcohol use, especially frequent drunkenness’ ([42], p. 146). Conversely, among adolescents above a certain age, non-drinking has been found to correlate with social isolation and self-perceived loneliness [45,46]. It has also been reported that experimental users of cannabis seem to be better off than non-users with respect to popularity and social adjustment [47,48].

All the studies above rely on the respondents' self-reported friendship relations or lack of them, but a more crucial issue is whether there is a positive correlation between the number of people who perceive the same person as their friend and that person's extent of substance use. A national survey of US secondary school students revealed that this was indeed the case with respect to drinking, but the number of received friendship nominations was unrelated to cannabis use [49]. However, a similar study of Scottish youth found that alcohol and drug use both correlated with the frequency of being perceived as a friend by peers [50]. Hence, it is unwarranted to interpret self–friend discrepancies with respect to substance as unequivocal evidence of norms misperception.

### Complex questions—valid responses?

While it may be difficult to give correct information about one's own drinking, it is undoubtedly harder to estimate others'—notably when the ‘others’ refer to a distal social group. If taken seriously, the items used to assess these perceptions require a substantial amount of cognitive effort. Thus, even for the brightest respondents, it is no small challenge to give reasonably accurate answers to questions such as ‘How much alcohol, on average, does a typical student at your university drink each day of a typical week?’[51] or to calculate the typical number of drinks consumed by age-mates at the national level on a night out on the pub or a club [12]. To estimate the percentage of co-students of the same age and gender who have vomited because of their drinking in the last 3 months [9] is also no simple task.

In their meta-analytical study, Borsari & Carey [4] speculated whether the observed self–other discrepancies with respect to drinking ‘may be a result of challenging questions rather than a genuine misperception of norms’ ([4], p. 337). It should thus be noted that the examples above are not exceptional. In fact, the first question cited stemmed from a version of the Drinking Norms Rating Form (DNRF) [2], which is an assessment tool that several studies in this field have applied.

In addition to a high level of complexity, the perceived meaning of the DNRF items and other similar items may be problematic. Thus, when asked about other students' average alcohol consumption on *each separate day* in a typical week, respondents may infer erroneously that daily drinking is common—otherwise, the question would have been framed differently. These kinds of questions might also mislead the survey participants to believe that they are supposed to estimate the consumption level only among the drinkers. Moreover, when making judgements under uncertainty, the respondents may assume that the values in the middle range of the response scale reflect the average or the usual frequency in the population [52,53].

The ‘typical student’ is probably the most common reference group in studies on perceived drinking norms. How this term is interpreted is evidently essential. From the researchers' perspective, it is synonymous with the average student but it is generally well established that the numerical meaning of vague quantifiers such as ‘typical’ or ‘usual’ may not be perceived as intended [54,55]. For instance, some respondents may have the stereotypical student or the most prevalent category of students with respect to drinking in mind when estimating how much the ‘typical student’ drinks and thus fail to take the abstainers and the light drinkers into account.

Numerous studies on cognitive aspects of survey methodology have demonstrated that the questions may shape the answers [52–56]. This important issue has barely been addressed in studies on self–other differences with respect to substance use. However, it has been reported that item specificity seems to matter. For instance, Larimer *et al*. [32] revealed that college students' perceived drinking norm for ‘the typical student’ was significantly higher than that for the typical student at the same gender, ethnicity and residence as the respondent. Such findings may in part reflect a proximity effect, but Borsari & Carey [4] showed that item specificity with respect to drinking behaviour also made a difference. The more specific the measures on own and peer alcohol use, the smaller was the observed tendency to overestimate. However, a majority of the 66 studies on perceived descriptive norms in their meta-analysis were based on relatively vague questions. Thus, on a scale from 0 (highly specific) to 100 (very vague), the average emerged as 64.

Unclear questions may also yield high rates of missing data, but item non-response is often not reported in studies on self–other differences with respect to substance use. Moreover, almost none of these studies have offered ‘I don't know’ as a response option. Wechsler & Kuo's [57] national survey of US college students is an exception in this respect. When asked to estimate the percentage of binge drinkers at their school, 12% answered that they did not know. The lower the personal level of drinking, the higher was the prevalence of such answers. Data from the Norwegian youth survey also shed some light on the issue. More precisely, when asked whether it was true or false that ‘about 50% of all 15–16-year-olds in Norway have used cannabis’,^1^ 38% of the 15–16-year-olds answered that they did not know. The vast majority (95%) of these respondents reported no personal use of cannabis, and in line with Weschler & Kuo's [57] findings, their prevalence of ‘I don't know’-answers (42%) far exceeded that observed among those who had tried the drug (18%) (*P* < 0.0001).

The social norms theory [5,16,17] and the literature on norms misperception more generally seem to rely on an assumption that individuals hold beliefs about other's involvement in normatively regulated behaviours. However, the findings above may be taken as an indication that quite a few young people, notably those who have little experience with substances, have no or only very vague pre-existing assumptions when responding to items on their peers' drinking or drug use. However, no study to my knowledge has examined how strongly young people believe that their estimates of peer substance use are correct.

### Underestimation and ‘accurate’ responses

Many studies have only reported the mean values for the key measures, implying that information about the prevalence of norms misperception and apparently correct responses is unavailable. Some of the studies that provide such information suggest that it may be common to underestimate peer drinking [10,57–60]. For instance, this was the case for nearly half the early adolescent respondents in a study by Juvonen *et al*. [59], while Franca *et al*. [60] reported that the proportion of college students who underestimated was higher than the proportion whose estimates exceeded the ‘actual’ drinking level. The latter study also indicated that quite a few held reasonably accurate beliefs about the issue.

Data from the Norwegian youth survey added to the picture. The senior high school students in the sample were asked to consider all same-aged students in their municipality and to report on a pre-coded scale the percentage that they believed had used cannabis. Fourteen per cent reported personal use of the drug, but a solid majority (67%) assumed that the prevalence was somewhere between 0% and 10%. Fewer than one in five (18%) believed that the prevalence exceeded 20%, implying that the occurrence of ‘correct’ answers was 15%.

As in other surveys, the above findings may have been affected by methodological limitations, but it should be kept in mind that full cohorts of students were assessed, that the response rate was high (87%) and that the question on peers' cannabis use was simple and presumably easy to understand. However, the results from this study and from other similar studies should never be taken at face value, among other things because they depend critically on characteristics of the key variables. The more finely graded the response scale for these variables, the lower the occurrence of ‘accurate’ perceptions and the higher the occurrence of apparently misperceived norms. In a sense, the magnitude of the ‘problem’ may thus be a matter of choice.

### The troublesome social comparison context

Some studies on perceived drinking norms have asked the respondents directly whether they believe that others drink more or less than themselves (e.g. [1,9,11]), but a far more common strategy is to assess such self–other differences indirectly. Thus, the standard procedure is to include two separate questions on the respondents' own substance use and on their perceived substance use by peers in the same questionnaire.

However, when respondents compare themselves with others or when they are likely to infer that such comparisons will be made, their responses may be affected by a self-serving bias. In other words, a social comparison context may promote individuals' tendency to see themselves as ‘better’ than other people or, alternatively, others may be seen as ‘worse’ than they otherwise would have been. Melson *et al*. [61] pursued this issue recently in a study on adolescent drinking and found support for the latter. More precisely, the respondents were far more likely to report that their peers consumed alcohol when they also were asked questions about their own consumption, but the prevalence of self-reported drinking did not vary with questionnaire type.

The findings above raise serious concerns about the research literature on misperceived norms in relation to substance use. The title of Melson and co-workers' [61] paper illustrates the point: ‘Overestimation of peer drinking: error of judgment or methodological artifact?’. However, it should be noted that while the discrepancy between the ‘actual’ and the perceived prevalence of drinking decreased significantly when the data were collected separately, rather than simultaneously, it did not disappear.

## CONCLUDING REMARKS

According to both literature reviews [3,5,6] and meta-analyses [4] it is well documented that young people tend to overestimate the extent of peer drinking and drug use. However, based on a critical examination of the literature in question, my conclusion is that the phenomenon has been exaggerated while the uncertainty surrounding the findings has been understated.

Several limitations characterize this research, including low response rates and widespread use of convenience samples, as well as the presence of contextual factors and the use of questionnaire items that may have created a bias in favour of ‘demonstrating’ that youth overestimate peer substance use. Conversely, although most studies conclude that the modal tendency is to overestimate, high levels of underestimation have also been observed. There is also suggestive evidence that young people do not necessarily possess preformed beliefs when responding to questionnaire items about peer substance use. Data from the Norwegian youth survey added to this picture.

In some cases, results that have been taken as evidence of exaggerated drinking norms may well reflect real differences—between individuals and their perceived friends, or between the survey participants and the average co-student. In other cases, what Borsari & Carey [4] referred to as ‘a genuine misperception of norms’ may have been at work. How such misperceptions can be measured adequately is far from clear, but one basic prerequisite is that the respondents match the group chosen for comparison. To ensure that this is the case, one could select groups rather than individuals and collect the data in a group context. More precisely, members of established groups (e.g. study groups or school classes) could be gathered together, and in such a setting the comparison target could be specified as ‘the other survey participants in the room’. This approach would also imply that vague and ill-defined reference groups could be replaced by a highly specific and easily understandable alternative.

Another suggestion for future research is to examine the respondents' comprehension of the questions that have been applied in previous research. How the commonly used term ‘the typical student’ is perceived stands out as particularly important. Evidently, whether—and if so, to what extent—young people believe ‘the typical student’ drinks more than themselves depends critically on their perceived meaning of this term. It is also possible that different subgroups of youth tend to interpret the same words and item formulations differently, and that this in part may explain why the extent of overestimation as well as magnitude of the observed self–other differences varies with the respondents' personal drinking level (e.g. [6–10,38]).

The issue raised by Melson *et al*. [61] should also be scrutinized further. Their innovative study showed that collecting data on the respondents' own drinking and on their perception of peer drinking at the same time may promote overestimation of the latter and the robustness of this finding should be tested. Moreover, I fully support the suggestion that ‘further research is warranted to examine more closely the potential active role of researcher-imposed methodologies in encouraging overestimation of young people's alcohol related perceptions’ ([61], p. 1083).

## Footnotes

1 According to nationally representative school surveys, the life-time prevalence of cannabis use among 15–16-year-olds in Norway was 9% in 2003 [62] and 6% in 2007 [63].
